# Incidence and Clinical Description of Lymphomas in Children and Adolescents with Vertical Transmission of HIV in Rio de Janeiro, Brazil, in Pre- and Post-Combined Antiretroviral Therapy Eras: A Multicentric Hospital-Based Survival Analysis Study

**DOI:** 10.3390/cancers14246129

**Published:** 2022-12-12

**Authors:** Nathalia Lopez Duarte, Ana Paula Silva Bueno, Bárbara Sarni Sanches, Gabriella Alves Ramos, Julia Maria Bispo dos Santos, Henrique Floriano Hess e Silva, Janaina de Oliveira Pondé, José Gilberto de Sá, Priscila Mazucanti Rossi, Patricia Regina Cavalcanti Barbosa Horn, Denise Cardoso das Neves Sztajnbok, Norma de Paula Motta Rubini, Cristiane Bedran Milito, Thalita Fernandes de Abreu, Marcelo Gerardin Poirot Land

**Affiliations:** 1 Faculty of Medicine (FM), Federal University of Rio de Janeiro (UFRJ), Rio de Janeiro 21941-617, Brazil; 2 Internal Medicine Postgraduate Program, Faculty of Medicine (FM), Federal University of Rio de Janeiro (UFRJ), Rio de Janeiro 21941-617, Brazil; 3 Transdisciplinary Center for Research in Child and Adolescent Health (NTISCA), Institute of Pediatrics and Childcare Martagão Gesteira (IPPMG), Federal University of Rio de Janeiro (UFRJ), Rio de Janeiro 21941-612, Brazil; 4 Pediatric Hematology Service, Institute of Pediatrics and Childcare Martagão Gesteira (IPPMG), Federal University of Rio de Janeiro (UFRJ), Rio de Janeiro 21941-612, Brazil; 5 Faculty of Medical Sciences (FCM), Pedro Ernesto University Hospital (HUPE), State University of Rio de Janeiro (UERJ), Rio de Janeiro 20551-030, Brazil; 6 Department of Infectious and Parasitic Diseases, Hospital Municipal Jesus (HMJ), Municipal Health Secretariat (SMS-RJ), Rio de Janeiro 20550-200, Brazil; 7 Department of Hematology, Pedro Ernesto University Hospital (HUPE), State University of Rio de Janeiro (UERJ), Rio de Janeiro 20551-030, Brazil; 8 Pediatric Infectious Diseases Division, Department of Pediatrics, Pedro Ernesto University Hospital (HUPE), State University of Rio de Janeiro (UERJ), Rio de Janeiro 20551-030, Brazil; 9 Department of Allergy and Immunology, School of Medicine and Surgery, Gaffrée and Guinle University Hospital (HUGG), Federal University of the State of Rio de Janeiro (UNIRIO), Rio de Janeiro 20270-004, Brazil; 10 Department of Pathology, Faculty of Medicine (FM), Clementino Fraga Filho University Hospital (HUCFF), Federal University of Rio de Janeiro (UFRJ), Rio de Janeiro 21941-617, Brazil; 11 Infectious and Parasitic Diseases Service, Institute of Pediatrics and Childcare Martagão Gesteira (IPPMG), Federal University of Rio de Janeiro (UFRJ), Rio de Janeiro 21941-612, Brazil

**Keywords:** HIV, vertical transmission (VT), combined antiretroviral therapy (cART), pediatric, lymphoma, epidemiology, incidence, Brazil

## Abstract

**Simple Summary:**

In Brazil, we have a considerable population of children living with HIV (CLWH), who are at a high risk of cancer development due to HIV infection (especially lymphomas), despite treatment with combined antiretroviral therapy (cART). This study aimed to learn how we might prevent these malignancies. We found a higher incidence of AIDS-defining malignancies (ADM) compared to non-AIDS-defining ones (NADM). In addition, ADM incidence rate decreased through widespread cART use. This study demonstrates the incidence of lymphoma in CLWH in Rio de Janeiro (RJ), Brazil, as well as the benefit of cART in reducing ADM and death occurrences in the Post-cART Era.

**Abstract:**

The incidence of cancer in children living with HIV (CLWH) is high and lymphomas are the most common type of cancer in this population. The combined antiretroviral therapy (cART) changed the natural history of HIV infection. To determine the incidence and profile of these CLWH malignancies in Rio de Janeiro (RJ), Brazil, we conducted a retrospective and observational study of vertically infected CLWH, ranging from 0–20 incomplete years, from 1995 to 2018, at five reference centers. The study period was divided into three eras in accordance with the widespread use of cART in Brazil. 1306 patients were included. Of the 25 lymphomas found, 19 were AIDS-defining malignancies (ADM); 6 were non-AIDS-defining malignancies (NADM). The incidence rate (IR) of lymphoma developing was 1.70 per 1000 children-year (95% CI 1.09–2.50). ADM development IR decreased from 2.09–1.75–0.19 per 1000 children-year (*p* < 0.001) through cART eras. Cumulative Nelson–Aalen hazards of developing ADM over a 20-year period were 3.73% in the Early-cART era, 3.07% in the Mid-cART era, and 0.32% in the Late-cART era (*p* = 0.013). This study demonstrates the IR of lymphoma in CLWH in RJ, Brazil, as well as the benefit of cART in reducing ADM and death occurrence in the Post-cART era.

## 1. Introduction

The incidence of cancer in children living with HIV (CLWH) is higher when compared to the general population [1,2,3], as also described for HIV-infected adults [4,5,6,7]. This higher occurrence is essentially due to immune system impairment resulting from the progressive depletion of CD4+ lymphocytes, loss of immune functions, persistent inflammation, chronic immune activation, and complex interactions with other oncogenic viruses (such as Epstein-Barr Virus (EBV) and Human Herpes Virus type 8 (HHV8)) and, ultimately, premature aging [8,9,10,11,12,13].

B-cell Non-Hodgkin Lymphoma is the most common group of cancer in CLWH, being classified as an AIDS-defining malignancy [14,15,16]. Lymphomas related to HIV infection are generally in an advanced stage, with extranodal involvement at the time of diagnosis, and typically suggest an aggressive clinical course [16,17].

Worldwide, 1.7 million children aged 0–14 years are HIV-infected and at an increased risk of developing cancer of which only 52% have access to antiretroviral therapy (ART) [18]. In 2021, 160,000 children were infected by HIV, 4000 of them in Latin America [19]. More specifically, in Brazil, we have about 6494 CLWH (ages 0–17) using cART and 848 reported cases of HIV in children and adolescents aged 0–19 years in 2021. For children under the age of five, the AIDS detection rate has been used as a proxy indicator for monitoring the vertical transmission (VT) of HIV. Thus, of the reported AIDS cases in children aged 0–13 years in 2021, 88.9% acquired the virus by VT [20].

The combined antiretroviral therapy (cART), which includes antiretroviral drugs from at least three different classes (e.g., protease inhibitors, nucleoside reverse transcriptase inhibitors, non-nucleoside reverse transcriptase inhibitors, and integrase inhibitors), or some two-drug based regimens, was thought to change the natural history of HIV infection [9,17,21,22]. It has been massively proved by previous studies that early cART access and maintenance of immune recovery during HIV infection remains a key strategy for the prevention of infection-related malignancies, including lymphomas [23,24].

The number of HIV-infected children receiving cART has been increasing since 1996. In Brazil, mono-and-dual antiretroviral therapy was initially provided in the early 1990s, and cART has been available since 1996, thus becoming the first developing country to provide free and universal access to cART [25,26,27]. This resulted in a substantial decrease in AIDS and mortality in HIV-infected children worldwide, as well as an increase in the survival of infected patients. On the other hand, there has been an increase in the prevalence of HIV in the pediatric population [22,28,29].

The impact of cART on cancer incidence in childhood and adolescence, as well as on the profile of malignant lymphoproliferative diseases in these populations, is broadly confirmed in many countries around the world, including some developing countries [22,28,30]. Nevertheless, in Brazil, little is known about the impact of cART use on the incidence of malignant lymphoproliferative diseases in CLWH, compared to the era before cART use.

Our study aimed to investigate the incidence of malignant lymphoproliferative diseases in HIV-vertically infected children and adolescents followed up in five reference institutions for the treatment of HIV/AIDS in the city of Rio de Janeiro, RJ, Brazil. The epidemiology of AIDS-defining malignancies (ADMs) and non-AIDS-defining malignancies (NADMs) in HIV-infected populations in Brazil, an important upper-middle-income country, has not been well described. To the best of our knowledge, this is the first pediatric study that aims to understand and analyze the impact of cART on the incidence and profile of malignant lymphoproliferative diseases in HIV-vertically infected patients in Brazil.

## 2. Materials and Methods

### 2.1. Study Design, Study Populations, and Data

For this multicentric, hospital-based and observational study of a retrospective cohort of pediatric patients, we included children aged one month to 19 years, 11 months, and 29 days of age with a confirmed diagnosis of HIV infection by VT, in accordance with the criteria of the Brazilian Ministry of Health/CDC [31], who started medical follow-up at one of the five participating institutions from 1 January 1995 to 1 January 2018.

The participating centers in the study were the following: Instituto de Puericultura e Pediatria Martagão Gesteira—IPPMG/UFRJ, Hospital Universitário Clementino Fraga Filho—HUCFF/UFRJ, Hospital Universitário Pedro Ernesto—HUPE/UERJ, Hospital Universitário Gaffrée e Guinle—HUGG/UNIRIO e Hospital Municipal Jesus—HMJ/SMS-RJ.

Data were collected by reviewing medical records to obtain information on medical evolution and results of relevant complementary exams. These also included data about the diagnosis of HIV infection and malignant lymphoproliferative disease, histopathologic evaluation, and clinical course until the beginning of malignant lymphoproliferative disease. Ethical approval was obtained from each institutional review board of the five participating centers.

### 2.2. Study Period Definition

We divided the study period from 1995 to 2018 into three subperiods in accordance with the widespread use of cART in CLWH in Brazil, as well as that which is described in international literature. Children and adolescents were classified into each era by the date of entrance into the participating institution: from 1995 to 1999 (1995–1999) for Early-period cART (Early-cART era), when the first protease inhibitors were introduced; from 2000 to 2003 (2000–2003) for Mid-period cART (Mid-cART era), when there was a simplification of cART and the introduction of non-nucleoside reverse transcriptase inhibitors (NNRTI) regimens; and from 2004 to 2018 (2004–2018) for Late-period cART (Late-cART era), when new cART regimens based on coformulation of antiretroviral drugs were initiated, increasing adherence and effectiveness of cART [9,17,22,29].

### 2.3. Tumor Samples and Laboratorial Analysis

The tumor samples found were reanalyzed and reclassified in accordance with the latest WHO classification, 2022, [32] through morphological and immunohistochemical analyses. All the analyses were performed at the Department of Pathology of the HUCFF/UFRJ.

### 2.4. Outcome Variables and Definition

Malignant lymphoproliferative disease diagnosis was reviewed by the study pathologist and reclassified following WHO 2022 criteria. The cases were defined by the ICD-11 MMS code for neoplasms of hematopoietic or lymphoid tissues. The codes for ADM used in this study are: 2A85.6 for Burkitt Lymphoma (BL) (13 cases) and 2A81.Z for Diffuse Large B-Cell Lymphoma (DLBCL) (6 cases). NADM was defined as patients who had an ICD-11 MMS code of 2B30.10 corresponding to Nodular Sclerosis Classical Hodgkin Lymphoma (NSCHL) (4 cases), 2A90.A for Anaplastic Large Cell Lymphoma (ALCL CD30+/ALK+) (1 case) and 2A90.C for Peripheral T-Cell Lymphoma (PTCL) (1 case) [14,16,33].

For the immunological status analysis of patients that developed malignant lymphoproliferative disease, the CDC category and immunosuppression stage (N/A/B/C and 1/2/3/unknown, respectively) were considered at the diagnosis date of neoplasm. For this study, CD4+ counts were used as a percentage which was available during the clinical follow-up period of referred patients. Counts above 25% define absence of immunosuppression (stage 1); between 15% and 25% define moderate immunodeficiency (stage 2); and below 15% define severe immunodeficiency (stage 3) [31]. Lastly, CD4+ values were considered with an arbitrary margin of 90 days (before or after) from the diagnosis date of malignant lymphoproliferative disease for the most accurate value.

### 2.5. Statistical Analysis

For numerical continuous variables, we calculated the measures of central tendency, i.e., means (standard deviation) or medians (interquartile range and range), depending on the variable’s distribution type (normal or not). We used the Kolmogorov–Smirnov test and the Shapiro–Wilk test for data normality. The Student *t* test was used to compare the means between groups. For variables that do not meet the requirements for normal distribution, we used the Mann–Whitney test for two groups and Kruskal–Wallis test for three or more groups. For categorical variables, the comparison of each group’s percentage or risk was evaluated by chi-square or Fisher’s exact test.

For survival analysis, the time of observation started at birth (Dynamic Cohort Time Zero) and ended at event occurrence or at the time of censoring. The outcome of interest was malignant lymphoproliferative disease occurrence (all lymphoma subtypes, ADM and NADM). The Median follow-up time was calculated using the Kaplan–Meier Curve.

The incidence rate (IR) of malignant lymphoproliferative diseases per 1000 children-year was calculated. The cumulative hazards of developing lymphoproliferative malignancies in the cohort in question were obtained by the Nelson–Aalen estimator. A *p*-value of less than 0.05 was used for statistical significance. The Hazard Ratio (HR) of individual predictors was calculated by the univariate Cox proportional hazard method.

The cumulative probability (CP) function of different outcomes (ADM, NADM, and Death) was calculated in the context of competing risks [34]. We also used the R Project “cmprsk” package (a free software environment for statistical computing and graphics), which implements the competing risk analysis [35].

In the case of non-convergence of the likelihood function, we used the R Project “coxphf” package which implements Firth’s penalized maximum likelihood bias reduction method for Cox regression. It has been shown to provide a solution in the case of monotone likelihood (non-convergence of likelihood function). The program fits profile penalized likelihood confidence intervals which were proved to outperform Wald confidence intervals [36].

Lastly, all data processing and statistical analysis were performed using the R Project version 4.0.2, and IBM SPSS Statistics software version 21.0.

## 3. Results

### 3.1. Characteristics of the Study Population and Incidence of Lymphoma in CLWH

We analyzed a total of 1306 HIV-vertically infected children: 624 (47.8%) were male, and 682 (52.2%) were female, with a global cohort median age of 12.41 years.

Of the 1306 patients, 25 children (1.91%) developed lymphoma. No other types of malignant lymphoproliferative diseases were found in the cohort. Of these 25 children, 14 (56%) were male, and 11 (44%) were female. Detailed medical records of the 25 patients were found. The main presenting features of the 25 patients are summarized in Table 1.

The calendar years of lymphoma diagnosis were as follows: 1996 (*n* = 01), 1998 (*n* = 02), 1999 (*n* = 02), 2000 (*n* = 03), 2001 (*n* = 02), 2002 (*n* = 04), 2004 (*n* = 04), 2005 (*n* = 03), 2007 (*n* = 01), 2010 (*n* = 02), 2013 (*n* = 01). When considering the date of birth as a defining parameter, the distribution was as follows: 1988 (*n* = 01), 1990 (*n* = 01), 1992 (*n* = 03), 1993 (*n* = 05), 1994 (*n* = 02), 1995 (*n* = 02), 1996 (*n* = 02), 1997 (*n* = 01), 1998 (*n* = 05), 1999 (*n* = 01), 2000 (*n* = 01), 2001 (*n* = 01).

Only two patients (numbers 14 and 23) had been exposed to prenatal antiretroviral therapy (AZT + DDI) starting at 32 weeks and 12 weeks, respectively. Only these children received antiretroviral therapy peripartum and for 28 days after birth (AZT).

The median age at the lymphoma diagnosis was 7.43 years (min–max 1.44–15.69 years; IQR 4.19). For ADM, the median age at the lymphoma diagnosis was 7.11 years (min–max 1.44–15.69 years; IQR 4.98). For NADM, the median age at the lymphoma diagnosis was 7.91 years (min–max 5.89–11.37 years; IQR 2.72).

The Median follow-up time for the global cohort was 12.63 years (12.25–13.01). Per era was also calculated. Early-cART: 12.86 years (12.34–13.39), Mid-cART: 13.51 years (12.93–14.08), Late-cART: 11.64 years (10.95–12.32).

### 3.2. Evolution of Lymphoma Diagnosis in HIV-Vertically Infected Children

For a better understanding of the behavior of these lymphoproliferative malignancies in CLWH we did the same incidence analysis for the whole group of lymphomas found in the cohort (25 cases) as well as for the two lymphoma groups—ADM (19 cases) and NADM (6 cases). The same analysis was performed for each cART era, taking the date of entrance into the participating institution as the defining parameter.

Table 2 shows the IR of lymphoma diagnosis (lymphomas per 1000 children-year) for the whole study period and for the three different eras, by sex and for ADM and NADM.

**Figure 1:**
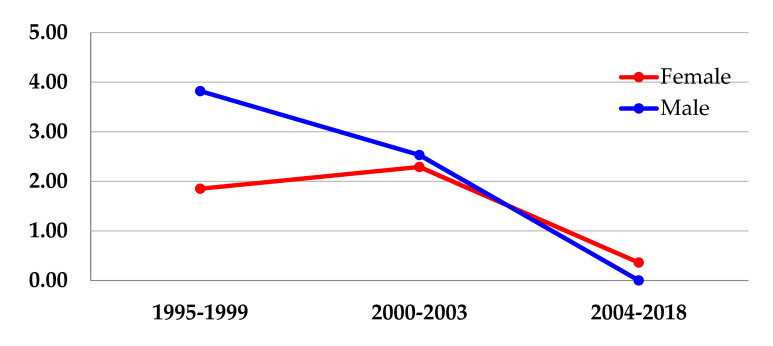
Incidence Rate (IR) of HIV-related Lymphoma for the Global cohort through Eras, by Sex.

The IR of lymphoma for the whole group was 1.70 per 1000 children-year (95% CI 1.09–2.50). Comparing the three calendar periods, we found that the IR of lymphoma diagnosis decreased (from 2.71–2.42–0.19 lymphomas per 1000 children-year; *p* < 0.001) through the cART eras. In the comparison by sex, we found that rates also decreased for both male and female through the eras (from 1.85–2.29–0.36 lymphomas per 1000 children-year for females, *p* = 0.001; from 3.82–2.53–zero lymphomas per 1000 children-year for males, *p* < 0.001) (Figure 1).

The IR of ADM diagnosis decreased (from 2.09–1.75–0.19 per 1000 children-year; *p* < 0.001) through the cART eras. It ranged from 0.74–1.83–0.36 lymphomas per 1000 children-year for females, *p* = 0.599, being non-significant; and decreased from 3.82–1.68–zero lymphomas per 1000 children-year for males, *p* < 0.001 (Table 2).

The IR of NADM diagnosis also decreased (from 0.63–0.65–zero per 1000 children-year; *p* < 0.001) through the cART eras. It decreased from 1.10–0.45–zero lymphomas per 1000 children-year for females, *p* < 0.001; and ranged from zero–0.84–zero lymphomas per 1000 children-year for males, *p* = 0.728, being non-significant) (Table 2).

**Figure 2:**
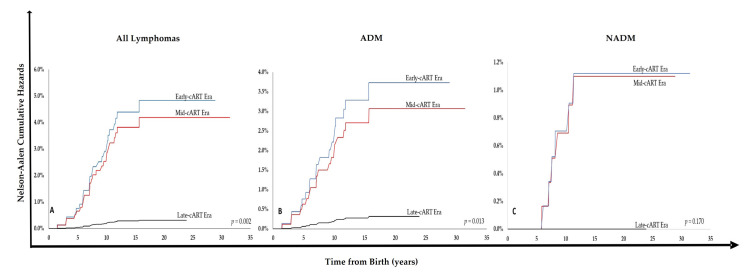
Nelson–Aalen Cumulative Hazards of Developing Lymphoma for HIV-vertically Infected Children per Era for all Lymphoma subtypes (**A**), ADM (**B**), and NADM (**C**).

The cumulative hazard of developing lymphoma for the global cohort in 20 years was 2.95%. Per era, it was 4.83% in Early-cART era, 4.20% in Mid-cART era, and 0.31% in Late-cART era (*p* = 0.002). For ADM, per era, it was 3.73% in Early-cART era, 3.07% in Mid-cART era, and 0.32% in Late-cART era (*p* = 0.013). Finally, for NADM, it was 1.10% in Early-cART era, 1.12% in Mid-cART era, and zero in Late-cART era, but non-significant (*p* = 0.17) (Figure 2).

The HR for developing lymphoma between Early and Late-cART eras was 15.41 (CI = 2.01–117.83; *p* = 0.008) and between Mid and Late-cART eras was 13.38 (CI = 1.73–103.70; *p* = 0.013). For developing ADM, the HR between Early and Late-cART was 11.85 (CI = 1.52–92.60; *p* = 0.018) and between Mid and Late-cART was 9.76 (CI = 1.22–78.03; *p* = 0.032). Finally, the HR for developing NADM between Early and Late-cART was 1.33 (CI = 0.21–8.64; *p* = 0.627) and between Mid and Late-cART was 1.54 (CI = 0.24–10.02; *p* = 0.757), being non-significant in both analyses.

### 3.3. Competing Risk of Developing Lymphoma and for Death Not Related to Lymphoma per Eras

**Figure 3:**
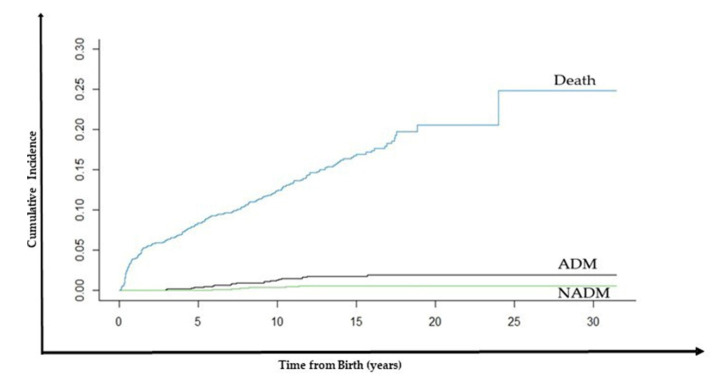
Cumulative Incidence (Competing Risk Model) for Death not related to Lymphoma or for ADM or NADM development in 30 years of evolution.

The competing risk of developing lymphoma and for death not related to lymphoma in 20 years was calculated (Figure 3). For the global cohort, the competing risk was 1.97% (CI = 1.50–2.90%) for ADM group, 0.60% (CI = 0.12–1.09%) for NADM group and 20.60% (CI = 17.26–23.91%) for death (Table 3).

Per era, the competing risk of developing ADM was 3.15% (CI = 1.09–5.20%) in Early-cART, 2.57% (CI = 0.80–4.33%) in Mid-cART, and 0.31% (−0.30–0.93%) in Late-cART era. For NADM, it was 0.88% (CI = −0.11–1.89%) in Early cART, 0.96% (CI = −0.12–2.06%) in Mid-cART, and zero in Late-cART era. Finally, for death, it was 29.59% (CI = 23.70–35.49%) in Early-cART, 18.08% (CI = 13.45–22.70%) in Mid-cART, and 14.48% (CI = 8.03–20.94%) in Late-cART era (Table 3)

Table 4 shows the HR for developing ADM or NADM between eras and for death not related to lymphoma, calculated by competing risk. For developing ADM, the HR between Early and Late-cART was 10.40 (CI = 1.33–81.60; *p* = 0.026), and between Mid and Late-cART was 9.48 (CI = 1.18–76.30; *p* = 0.035). For death, the HR between Early and Late-cART was 3.09 (CI = 2.12–4.71; *p* < 0.001), and between Mid and Late-cART was 1.72 (CI = 1.13–2.62; *p* = 0.011). For NADM, it was not possible to calculate the HR as the data did not converge since there was a division by zero.

### 3.4. Subtypes of Lymphomas Found in the Cohort

The survey showed that 25 children developed lymphoma as malignant lymphoproliferative disease, being 21 Non-Hodgkin Lymphoma (NHL), and 4 Hodgkin Lymphoma (HL). Of the NHL group, we had 13 BL, 6 DLBCL, 1 ALCL CD30+/ALK+, and 1 PTCL. Of the HL group, we had 4 NSCHL. No patient experienced more than one event.

According to Table 1, 19 (76%) of the 25 lymphomas were B-cell NHL. Only one patient had CNS involvement (BL), which was not primarily nodal. There was diffuse abdominal involvement in eight, small intestine in five, bone in five (jaw, facial bones, and femur), mediastinum in four, liver in four, bone marrow (BM) in four, gallbladder in two, kidney in two, ovary in two, soft palate in one, parotid in one, axillary lymph node in one, lung in one, and stomach involvement in one. Some cases had more than one site of involvement.

Three patients were not evaluated for CNS involvement and two patients were not available for BM involvement as analyses were not performed during the treatment period.

For HL (16% of the lymphoma cases), all of them NSCHL, one had cervical and thoracic lymph node involvement, one had mesenteric lymph node involvement, one had BM involvement, and one had diffuse lymph node involvement (cervical, thoracic, mesenteric, and inguinal) besides spleen and small intestine involvement.

Finally, for T-cell NHL (8% of the lymphoma cases), one case (ALCL CD30+/ALK+) had submandibular lymph node involvement as well as diffuse abdominal involvement (spleen, small intestine, and mesenteric lymph nodes). The other case (PTCL) had mediastinum and BM involvement.

**Figure 4:**
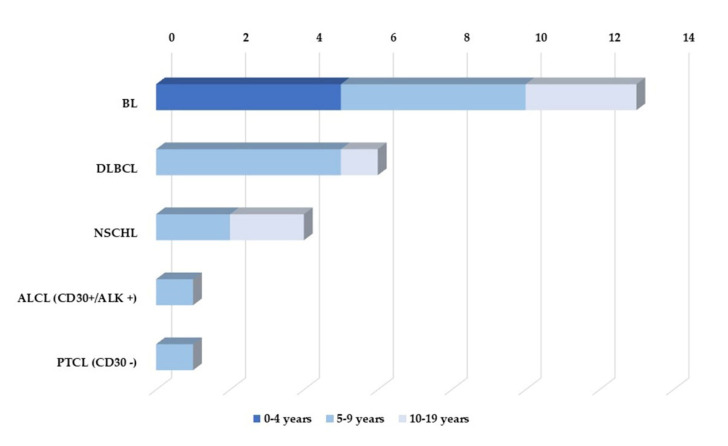
Lymphoma subtypes found in the cohort according to Age group at diagnosis. Abbreviations: Anaplastic Large Cell Lymphoma (ALCL CD30+/ALK+); Burkitt Lymphoma (BL); Diffuse Large B-Cell Lymphoma (DLBCL); Nodular Sclerosis Classical Hodgkin Lymphoma (NSCHL); Peripheral T-Cell Lymphoma (PTCL).

B-cell NHL occurred at a median age of 7.11 years (range, 1.44–15.69 years; IQR: 4.98). HL occurred at a median age of 8.82 years (range, 5.89–11.37 years; IQR: 3.94). For T-cell NHL, we only had two cases, at 7.62 (ALCL CD30+/ALK+) and 8.20 (PTCL) years. Figure 4 illustrates the different lymphoma subtypes found in the cohort according to age group at diagnosis.

In most patients that developed lymphoma (16 of 25), HIV infection was fully symptomatic with overt immunodeficiency (CDC C3 subcategory). A total of 3 of the 25 cases, all included in the C category, could not be evaluated for CD4+ T-cell counts categories, as patients had not performed CD4+ counts in the eligible period for the study (3 months before/3 months after lymphoma diagnosis). The CD4+ count and the CDC classification at the time of lymphoma diagnosis are reported in Table 1.

In 5 cases, B-cell NHL occurred in children without severe immunosuppression (CDC stage 1 or 2), and in 11 cases, with severe immunosuppression (CDC stage 3). A total of 3 of the 19 cases, all of them included in the C category, could not be evaluated as patients had not performed CD4+ counts.

Regarding HL, three of four cases occurred in children with severe immunosuppression (CDC stage 3). Both T-cell NHL also occurred in children with severe immunosuppression (CDC stage 3).

The lymphoma IR for immunologic CDC stage 3 (16 patients) was 1.08 per 1000 children-year (95% CI, 0.62–1.76), whereas for stages 1 and 2 (6 patients), it was 0.40 per 1000 children-year (95% CI, 0.14–0.88). For the three patients without CD4+ counts, the lymphoma IR (all in category C) was 0.20 per 1000 children-year (95% CI, 0.04–0.59).

## 4. Discussion

To the best of our knowledge, this is the first Brazilian study reflecting the effects of cART on the incidence and morphological characterization of lymphomas in an HIV-vertically infected pediatric cohort, as well as on the survival of this population.

Producing reliable cancer incidence estimates in HIV-infected pediatric population is a challenge. Sometimes HIV cohorts may not record cancer cases, and hospital-based cancer registries may not record HIV infection status. Furthermore, Rio de Janeiro does not have a population-based registry for CLWH (nor other Brazilian states) or for cancer.

Brazil is presumably considered a model for the world in terms of public response to the AIDS epidemic and has offered a free supply of ART and medications for opportunistic diseases through the public health system since the 1990s [25,37]. In 2015, Castilho et al. first described the cancer epidemiology of a Brazilian HIV-infected adult cohort in comparison to the general Brazilian population and to a US HIV-infected adult cohort. According to this study, cancer trends in adults living with HIV in Brazil are similar to those observed in high-income countries, where cART is also widely available. In other words, overall rates of ADMs appeared to be decreasing, while NADMs rates remained constant. Nevertheless, these findings must be confirmed in the pediatric population [38]. Other recent adult studies from Brazil have indicated that NADM increasingly contributes to morbidity and mortality in HIV-infected individuals [39]. However, the epidemiology of ADMs and NADMs in HIV-infected individuals has not been described in Brazil for both adult and pediatric populations.

Similar to previous studies, we verified that the IR of lymphoma diagnosis decreased throughout the cART eras [38,40]. The same behavior was seen by sex, with a higher incidence in males in the Early- and Mid-cART eras, in accordance with the literature [22,38,41]. In the Late-cART era, we had only one case of lymphoma in our population: a female one.

In our study, the IR of ADM diagnosis decreased markedly and progressively after the cART implementation, in line with findings from other pediatric and adult studies [6,22,38,42,43]. For NADM, we observed a slight increase in IR between the Early- and Mid-cART eras, and a substantial decrease in Late-cART, with zero cases in this period. However, it was not statistically significant.

The cumulative hazard of developing lymphoma in 20 years also declined, both for the general cohort and when considering ADM development. For the NADM, on the other hand, there was no statistical difference, probably due to the small number of cases observed in this part of the cohort. The reasons for the lower incidence of NADM in our cohort may be related to their younger age in relation to adult studies. Given that HIV infection is permanent, an even higher long-term risk for NADM development may yet be demonstrated in a cohort such as ours, and the importance of continued and possibly lifelong monitoring of HIV-vertically infected children throughout adulthood should be highlighted.

The competing risk analysis showed a significant downward trend in the CP of death not related to lymphoma and ADM development in our study population, which was not possible to determine for NADM. The CP adjustment for ADM was slightly lower when using competing risks analysis compared to the conventional Nelson–Aalen estimator. Both assays indicate that the beneficial effects of cART on immune recovery were critical to reduce the consequences of immunodeficiency in non-neoplastic death, possibly caused by opportunistic infections, and ADM development [44]. It was not possible to demonstrate the same beneficial effect for NADM, which may be explained by two hypotheses: either NADM is not related to immunodeficiency [45], or the small number of cases and the relatively short follow-up time were insufficient to verify a statistically significant decrease in incidence between the cART eras, as discussed before. It is known that the increased production of pro-inflammatory cytokines and immunosenescence due to HIV infection, ART toxicity, co-infections, in addition to the aging of this population and other particularities, may lead people living with HIV (PLWH) to develop mainly non-AIDS comorbidities nowadays, including NADMs [15,17,29,38]. We did not find another similar study that analyzed the competing risk of developing malignancies in a similar cohort to use as a comparison. This paper, therefore, presents another important piece of information. An important point to highlight is that the competing risk of death not related to lymphoma suffered an expressive reduction of about 50% through the eras. Nonetheless, the current risk of death is still high, which corroborates the low life expectancy of CWLH in Brazil compared to HIV-uninfected children [46,47], which can be related to the inferior socioeconomic indexes of this population, despite the good antiretroviral coverage provided by the Ministry of Health. Factors such as low adherence to cART, insufficient numbers of qualified caregivers in primary care units, low levels of family education, low neighborhood Human Development Index (HDI), among others, should be considered in this analysis. [22,30,48]. It is important to underline that, in the Post-cART era, cardiovascular and metabolic complications, in addition to other chronic diseases, have been the major cause of death in this population [10].

In our sample, we have 25 lymphoma cases. NHL was the most common lymphoma group (76%). It is known that B-cell NHL is a neoplasm group related to HIV immune suppression that mainly includes all aggressive B-cell NHL as BL and DLBCL [10,16]. In line with this, between NHL, BL and DLBCL were, respectively, the most frequent subtypes found in our study population. This lymphoma profile is already well-established by previous pediatric studies [40,44].

Besides the 21 NHL cases, we also had 4 HL cases. NSCHL is the most frequent HL subtype in PLWH and is also often associated with EBV infection [12]. Notably, all HL cases found in our cohort were NSCHL, and this finding is consistent with the EBV epidemiology in non-Caucasian-predominant ethnic groups and people living in developing and low-income countries [49].

Additionally, our cohort has two different subtypes of T-cell lymphomas, i.e., one ALCL CD30+/ALK+ and one PTCL. T-cell lymphomas are rare in HIV-positive individuals, comprising 3% of lymphomas in PLWH. Usually, they are diffuse, with bone marrow involvement, features also observed in our cases. According to Arzoo et al., patients with HIV-positive T-cell lymphomas have a similar prognosis to B-cell HIV-related ones [50,51].

Finally, it is essential to mention that other B-cell NHL subtypes frequently found in PLWH, such as Plasmablastic Lymphoma (PBL), Primary Effusion Lymphoma (PEL), and Primary Central Nervous System Lymphoma (PCNSL), were not found in our population.

As expected, HIV infection was fully symptomatic with overt immunodeficiency (C3 CDC subcategory) in most patients that developed lymphomas. The tumor IR for this group (16 patients) was 1.08 per 1000 children-year (95% CI, 0.62–1.76). In addition, B-cell NHL mainly occurs in children with severe immunosuppression. However, although it is known that HL is not related to immunosuppression promoted by HIV, being commonly found in patients with medium to higher CD4+ counts [16,50], in our cohort, 75% of the patients developed HL when in severe immunodepression. Finally, as also expected, both T-cell NHL cases occurred in a severe immunosuppression scenario. The association of severe CD4+ suppression with cancer is consistent with the attribution of the benefits of cART in the prevention of opportunistic cancers in the restoration of the adaptive immune response [12]. Findings of previous studies justify the recommendations to start children on cART as soon as they are diagnosed with HIV, regardless of their CD4+ immune status [52].

Lymphomas observed in this study were mostly diffuse and extranodal, bulky disease and had an early age of onset, which is in accordance with the literature [4,24]. B-cell NHL occurred at a median age of 7.11 years. Among the ADMs, BL was the only lymphoma subtype that occurred in children between 0 and 5 years of age, which is consistent with the aggressive profile of this neoplasm. HL occurred at a median age of 8.82 years, and T-cell NHL occurred in children with ages 7.62 (ALCL CD30+/ALK+) and 8.20 (PTCL). In this analysis, it is essential to consider the role of VT of HIV in developing these malignancies. The decision to analyze a pediatric cohort vertically infected by HIV was due to the possibility of knowing the exact date of infection by the virus and to an understanding of the exact time for the development of the neoplasm since the viral infection. Perinatally HIV-infected (perHIV) children have a higher risk of developing cancer due to immunosuppression and co-infections with oncogenic viruses. HIV pathogenesis differs in perHIV due to age-related differences in the immune system at time of infection, route of transmission, and cART start time. Indeed, all the variables mentioned above significantly impacted immune system development in perHIV, and the disease can have a faster progression [4,53]. This is because the immune system of newborns is still immature and unable to mount an efficient immune response, resulting in high levels of HIV viremia, which decreases with the introduction of cART [54]. This way, CLWH with vertically acquired infection showed a higher risk of ADM but a lower risk of NADM development [55]. As one of the expected effects of immune reconstitution promoted by cART, outcomes of HIV-infected cancer patients are known to be better in the Post-cART era [28,38,56,57].

Most lymphoma diagnoses occurred from 2004 to 2018. However, when considering the date of birth as a parameter, 23 of the 25 lymphoma cases found in the cohort were developed by CLWH born before the year 2000, with two cases born in the Mid-cART era and zero cases born in the Late-cART era. Although this observation was not fully analyzed in this paper, it could corroborate the carcinogenic potential of HIV and indicate the protective potential of cART in lymphoma development [58,59].

The strengths of our study include a large, multicentric, and retrospective cohort with high-quality pathologic diagnoses, a long calendar period covered, and a robust median time of follow-up. On the other hand, the limitations of this work are related to the fact that it is a hospital-based study, since there is not a population-based register for CWLH in Brazil, nor for cancer in general in Rio de Janeiro. In addition, it is a retrospective study with a 23-year follow-up. As such, access to medical records and tumor biopsies was difficult and their quality was also an issue. The loss of some tumor samples and the deterioration of others made it impossible to reclassify all samples according to new WHO 2022 guidelines, which occurred in five cases. Therefore, we took the reports given by qualified pathologists at the time of lymphoma diagnosis as a basis for the classification.

## 5. Conclusions

This study is based on a large and multicentric cohort of CLWH who attended five centers of excellence for HIV treatment in Rio de Janeiro, Brazil, and, for the first time, describes the impact of cART in lymphoma incidence in this population and also elucidates the subtypes of these cancers and its rates, as well as the risk of developing lymphoma and of death not related to lymphoma through cART eras. This study made it clear that cART is a remarkable disease-modifying therapy that reduces the incidence of ADM. It was not possible to determine cART effects in NADM. Therefore, starting cART as early as possible prevents severe CD4+ lymphocyte suppression, restores immunological surveillance and, in turn, can reduce the risk of ADM in CLWH, as well as the risk of death.

The WHO highlights the importance of early HIV screening and diagnosis for all children and adolescents for a better prognosis. It is critical that HIV treatment policies and programs such as routine maternal screening, prevention of VT, monitoring of exposed newborns, and early infant HIV diagnosis programs are maintained to prevent complications such as cancer development. Given the high number of PLWH and the massive number of new annual infections around the world, there is currently a recognized global need for improved screening, diagnosis, and treatment programs for cancer in HIV-infected populations. Despite the findings of this study, it is still unknown whether cancer trends in CLWH in Brazil are similar or not to those in high-income countries. This work was designed to improve the quality of HIV treatment in this population while we learn how we may prevent these malignancies. Finally, but with an important caveat, we did not find other types of neoplasms in this study population with a follow-up period of 23 years, including solid cancers. The reasons for this need to be clarified in future studies.

## Figures and Tables

**Table 1:** Presenting features of 25 HIV-Vertically Infected Children and Adolescents who developed Lymphoma.

Patient Index	Sex	Date of Diagnosis	Age (Years)	CDC Class	CD4+ (%)	Lymphoma Subtype	ICD-11 MMS	Site
1	M	23 January 1996	3.04	C2	18%	BL	2A85.6	Jaw, facial bones, and femur (R/L)
2	F	2 September 1998	5.89	C3	3%	NSCHL	2B30.10	Diffuse mesenteric lymph nodes
3	F	15 July 1999	5.35	C3	2%	BL	2A85.6	Gallbladder, femur (R/L), and BM
4	F	20 September 1999	1.44	C	NP	BL	2A85.6	Mediastinum and BM
5	M	31 March 2000	7.11	C3	2%	BL	2A85.6	Stomach, kidney (L), and diffuse abdominal mass
6	M	2 June 2000	11.59	C3	7%	BL	2A85.6	Femur (R), small intestine
7	M	3 October 2000	10.26	C3	21%	BL	2A85.6	Small intestine and BM
8	M	15 January 2001	7.01	C2	16%	DLBCL	2A81.Z	Parotid (R), jaw (R), and diffuse abdominal mass
9	M	24 September 2001	8.20	C3	15%	PTCL	2A90.C	Mediastinum and BM
10	F	21 February 2002	9.19	C3	9%	BL	2A85.6	Mediastinum, liver, ovary (R/L), and diffuse abdominal mass
11	F	26 August 2002	10.53	C3	21%	NSCHL	2B30.10	Cervical (R) and thoracic lymph nodes (R/L)
12	M	29 December 1998	4.75	C	NP	BL	2A85.6	Diffuse mesenteric lymph nodes, gallbladder, liver, kidney (L), and diffuse abdominal mass
13	M	10 February 2004	7.62	C3	14%	ALCL (CD30+/ ALK+)	2A90.A	Submandibular lymph node (L), diffuse mesenteric lymph nodes, spleen, and small intestine
14 *	F	5 January 2004	7.10	C1	36%	NSCHL	2B30.10	BM
15	M	24 January 2004	6.03	C2	25%	DLBCL	2A81.Z	Small intestine
16	F	19 July 2005	7.43	C3	9%	BL	2A85.6	CNS, liver, small intestine, and BM
17	M	17 August 2005	9.98	C2	18%	DLBCL	2A81.Z	Soft palate
18	M	18 October 2005	11.87	C3	NP	BL	2A85.6	Facial bones (L)
19	M	14 September 2010	9.54	C2	NP	BL	2A85.6	Mediastinum, liver, and diffuse abdominal mass
20	F	29 March 2007	11.37	C3	33%	NSCHL	2B30.10	Diffuse lymph node involvement (cervical (R), thoracic (R/L), mesenteric, and inguinal (R))
21	F	5 November 02	4.56	C3	NP	BL	2A85.6	Ovary (L) and diffuse abdominal/pelvic mass
22	M	15 August 2013	15.69	C3	18%	DLBCL	2A81.Z	Lung, mediastinum, and small intestine
23 *	M	25 March 2004	5.94	C3	NP	DLBCL	2A81.Z	Diffuse abdominal mass
24	F	3 May 2002	2.95	C	NP	BL	2A85.6	Diffuse abdominal mass
25	F	30 June 10	10.08	C3	2.6%	DLBCL	2A81.Z	Axillary lymph node (L)

Abbreviations: Anaplastic Large Cell Lymphoma (ALCL CD30+/ALK+); Bone Marrow (BM); Burkitt Lymphoma (BL); Central Nervous System (CNS); Diffuse Large B-Cell Lymphoma (DLBCL); Female (F); Nodular Sclerosis Classical Hodgkin Lymphoma (NSCHL); ICD-11 for Mortality and Morbidity Statistics (ICD-11 MMS); Male (M); Not Performed (NP); Peripheral T-Cell Lymphoma (PTCL). * These two patients received prenatal HIV-directed treatment and antiretroviral therapy peripartum.

**Table 2:** Summary of the Epidemiologic Trends of Lymphoma Diagnosis (Lymphomas per 1000 Children-Year) in HIV-Vertically Infected Children in Rio de Janeiro, Brazil, from 1995 to 2018.

	1995–2018		1995–1999		2000–2003		2004–2018	
	*n*	Rate (95% CI)	*n*	Rate (95% CI)	*n*	Rate (95% CI)	*n*	Rate (95% CI)
Total of lymphomas in cohort	25	1.70 (1.09–2.51)	13	2.71 (1.44–4.64)	11	2.42 (1.21–4.32)	01	0.19 (0.005–1.04)
F	11	0.75 (0.37–1.34)	05	1.85 (0.60–4.32)	05	2.29 (0.74–5.35)	01	0.36 (0.01–2.00)
M	14	0.95 (0.52–1.60)	08	3.82 (1.65–7.53)	06	2.53 (0.93–5.51)	Zero	Zero
ADM	19	1.29 (0.78–2.01)	10	2.09 (1.00–3.84)	08	1.76 (0.76–3.46)	01	0.19 (0.005–1.04)
F	07	0.48 (0.19–0.98)	02	0.74 (0.09–2.67)	04	1.83 (0.50–4.70)	01	0.36 (0.01–2.00)
M	12	0.82 (0.42–1.42)	08	3.82 (1.65–7.53)	04	1.69 (0.46–4.32)	Zero	Zero
NADM	06	0.41 (0.15–0.89)	03	0.63 (0.13–1.83)	03	0.66 (0.14–1.93)	Zero	Zero
F	04	0.27 (0.07–0.70)	03	1.10 (0.23–3.24)	01	0.46 (0.01–2.55)	Zero	Zero
M	02	0.14 (0.02–0.49)	Zero	Zero	02	0.84 (0.10–3.05)	Zero	Zero

Abbreviations: AIDS-defining malignancies (ADM); Confidence Interval (CI); Female (F); Male (M); Non–AIDS-defining malignancies (NADM).

**Table 3:** Competing Risk for Death not related to Lymphoma or for ADM or NADM development in 20 years of evolution.

Era	Outcome	Competing Risk CI (95%)	Outcome	Competing Risk CI (95%)	Outcome	Competing Risk CI (95%)
Global cohort	ADM	1.97% (1.50–2.90%)	NADM	0.60% (0.12–1.09%)	Death	20.60% (17.26–23.91%)
Early-cART	ADM	3.15% (1.09–5.20%)	NADM	0.88% (−0.11–1.89%)	Death	29.59% (23.70–35.49%)
Mid-cART	ADM	2.57% (0.80–4.33%)	NADM	0.96% (−0.12–2.06%)	Death	18.08% (13.45–22.70%)
Late-cART	ADM	0.31% (−0.30–0.93%)	NADM	Zero	Death	14.48% (8.03–20.94%)

Abbreviations: AIDS-defining malignancies (ADM); Combined Antiretroviral Therapy (cART); Confidence Interval (CI); Non–AIDS-defining malignancies (NADM).

**Table 4:** Hazard Ratio (HR) for Death not related to Lymphoma or for ADM or NADM development between Eras (Competing Risk analysis).

	ADM		NADM *		Death	
	Hazard Ratio IC (95%)	*p*-Value	Hazard Ratio IC (95%)	*p*-Value	Hazard Ratio IC (95%)	*p*-Value
Early-cART	10.40 (1.33–81.60)	0.026	NA	NA	3.09 (2.12–4.71)	<0.001
Late-cART	1				1	
Mid-cART	9.48 (1.18–76.30)	0.035	NA	NA	1.72 (1.13–2.62)	0.011
Late-cART	1				1	

Abbreviations: AIDS-defining malignancies (ADM); Combined Antiretroviral Therapy (cART); Confidence Interval (CI); Non–AIDS-defining malignancies (NADM). * For NADM, it was not possible to calculate Hazard Ratio (HR) since data did not converge.

## Data Availability

The data presented in this study are available on request from the corresponding author.
